# Vitreous hyper-reflective dots in pseudophakic cystoid macular edema assessed with optical coherence tomography

**DOI:** 10.1371/journal.pone.0189194

**Published:** 2017-12-15

**Authors:** Wilfried Glatz, Gernot Steinwender, Lisa Tarmann, Eva Maria Malle, Marlene Schörkhuber, Werner Wackernagel, Goran Petrovski, Andreas Wedrich, Domagoj Ivastinovic

**Affiliations:** 1 Department of Ophthalmology, Medical University of Graz, Graz, Austria; 2 Centre of Eye Research, Department of Ophthalmology, and Norwegian Center for Stem Cell Research, Oslo University Hospital, University of Oslo, Oslo, Norway; University of Michigan, UNITED STATES

## Abstract

**Purpose:**

This study compares the presence of vitreous hyper-reflective dots (VHDs) detected with optical coherence tomography (OCT) between eyes with pseudophakic cystoid macular edema (CME) and those with no CME after cataract surgery. In addition, we evaluated the impact of VHDs on the responsiveness of pseudophakic CME to cortisone treatment.

**Setting:**

Department of Ophthalmology, Medical University of Graz, Austria.

**Design:**

Retrospective, monocenter case-controlled study.

**Methods:**

Inclusion criteria for the study group and the control group were CME and no CME within 12 weeks following uneventful phacoemulsification in otherwise healthy eyes, respectively. VHDs (number and size) and the macular thickness were assessed with OCT. Furthermore, the number of peribulbar or intravitreal steroid injections was assessed.

**Results:**

A total of 284 eyes from 267 patients were analyzed, among which 119 met the inclusion criteria for the study (n = 63) and the control group (n = 56). VHDs were observed in 54 (85.7%) study eyes and 21 (37.5%, p = 0.013) control eyes. The number of VHDs was 3.9±3.4 in the study group and 0.7±1 in the control group (p<0.001). The size of the VHDs was 33.5±9.1 μm and 36.6±17.9 μm in the study and control groups, respectively (p = 0.978). Overall, the number of VHDs correlated with central subfield thickness (r = 0.584, p<0.001), cube volume (r = 0.525, p<0.001), and postoperative best-corrected visual acuity (BCVA) (r = -0.563, p<0.001). The number of VHDs did not correlate with the frequency of peribulbar or intravitreal steroid injections.

**Conclusion:**

VHDs occurred more often in eyes with CME than in eyes without CME following cataract surgery. In addition, the number of VHDs had an impact on the extent of macular thickening and subsequently postoperative BCVA. No correlation was found between the number of VHDs and the frequency of required peribulbar or intravitreal steroid injections.

## Introduction

Cystoid macular edema (CME) after uneventful cataract surgery was reported to occur in 0.2–2.35% of eyes before optical coherence tomography (OCT) was routinely used for its assessment [1–4]. Using OCT, however, revealed significantly higher rates of pseudophakic CME in up to 13.9% of eyes [2,5–8]. The reason for its occurrence is the breakdown of the blood-retina barrier and the postoperative release of inflammatory mediators and up-regulation of pro-inflammatory genes and proteins in the retina [9–11]. The existence of vitreo-macular traction has also been considered a confounding factor in CME development [7].

Optical coherence tomography (OCT) provides objective and quantitative evaluation of the retina without invasiveness and ethical concerns [12,13]. The evolution of OCT technology has facilitated the assessment of structures adjacent to the retina, including the choroid and the vitreous [8,14–17]. Recently, Oh et al. detected hyper-reflective dots in the preretinal vitreous of patients who underwent cataract surgery [8]. The nature of these vitreous hyper-reflective dots (VHDs) is unknown; however, it has been assumed that they originate from lens fragments or represent conglomeration of inflammatory cells, or epithelial cells released from the ciliary epithelium due to surgical trauma or perhaps denatured collagen fibrils [18–21]. Oh et al. found a significant relationship between VHDs and pseudophakic CME, which suggests the involvement of VHDs in the postoperative inflammatory process following phacoemulsification [8]. However, their conclusions are based on the observation of a significantly higher number of VHDs following cataract surgery in only 10 eyes with CME compared to 62 eyes with no CME after the surgery. The aim of our study is to evaluate the association between VHDs and CME by assessing their presence and number in a larger cohort of eyes with CME after cataract surgery. Therefore, eyes with confounding factors that might have contributed to the occurrence of CME including posterior capsule rupture, previous surgeries, diabetes, uveitis etc. were excluded [2]. The second goal is to evaluate the impact of VHDs on the responsivity of pseudophakic CME to cortisone treatment.

## Methods

This is a retrospective case-control study approved by the ethics committee of the Medical University Graz, Austria. The study adhered to the tenets of the Declaration of Helsinki. The researchers of this study had access to the data, which was saved in an anonymized data bank. There was no written informed consent provided, because the ethics committee waived the requirement for informed consent due to the design of the study. We reviewed the medical records of patients who presented with CME following cataract surgery at our hospital between January 2013 and August 2015. Spectral domain OCT was conducted with OCT Spectralis version 6.0.9 software (Heidelberg Engineering, Heidelberg, Germany) using volume scanning with 25 sections covering a field of 20x20° in the macular region. The device used a bandwidth of 297 nm and a wavelength of 815 nm. [14,22]. A built-in eye tracking software (TruTrack) ensured exact position of the recorded scans [14]. Sections were received using the high-speed mode with a resolution of 7 μm axially x 14μm laterally and a distance of 240μm between sections [22]. CME was defined as macular thickness >300 μm and the presence of intraretinal hypo-reflective cysts within the ETDRS (Early Treatment Diabetic Retinopathy Study) circle [2,8,23]. Inclusion criteria were post-phacoemulsification occurrence of CME within 12 weeks after the surgery for the study group and no CME occurrence within 12 weeks postoperatively for the control group. The control group consisted of pseudophakic eyes with no CME in which OCT was performed in the course of another study at 4, 8 and 12 weeks after surgery. The age and the follow-up of study eyes and control eyes were matched as closely as possible. Exclusion criteria for both groups were capsule rupture with or without anterior vitrectomy; previous interventions including vitrectomy, glaucoma surgery or Nd:YAG laser capsulotomy; exudative or dry age-related macular degeneration; presence of diabetes; history or presence of retinal vein occlusion or uveitis; macular pucker; inappropriate quality of the OCT image (quality score <20); or incomplete data. The quality score of OCT scans was automatically assessed for each OCT scan and displayed on top of the scan. The quality of an OCT scan is potentially biased by segmentation failure or a low signal strength due to media opacities, small pupil diameter, ocular surface disease, or blinking during examination. According to the instruction manual of the manufacturer, a quality score of ≥20 was considered as sufficient.

Prior to surgery, all of the patients underwent detailed preoperative ophthalmic examination including best-corrected visual acuity (BCVA); biomicroscopy with indirect ophthalmoscopy; applanation tonometry; and biometry including keratometry values, axial length and the anterior chamber depth (IOL-Master 500, Carl Zeiss Meditec, Jena, Germany). Axial length was measured from the corneal vertex to the retinal pigment epithelium by partial coherence interferometry. In case of dense cataract, axial length values were obtained by ultrasound (Axis II PR, Quantel Medical, Clermont-Ferrand, France), measuring the distance between the corneal vertex and the inner limiting membrane of the retina. All patients underwent cataract surgery under retrobulbar anesthesia using the phacoemulsification technique for the removal of the crystalline lens and subsequent implantation of a monofocal hydrophobic acrylic intraocular lens in the capsular bag. At the end of the surgery, 0.1 ml cefuroxime was injected into the anterior chamber.

**Fig 1:**
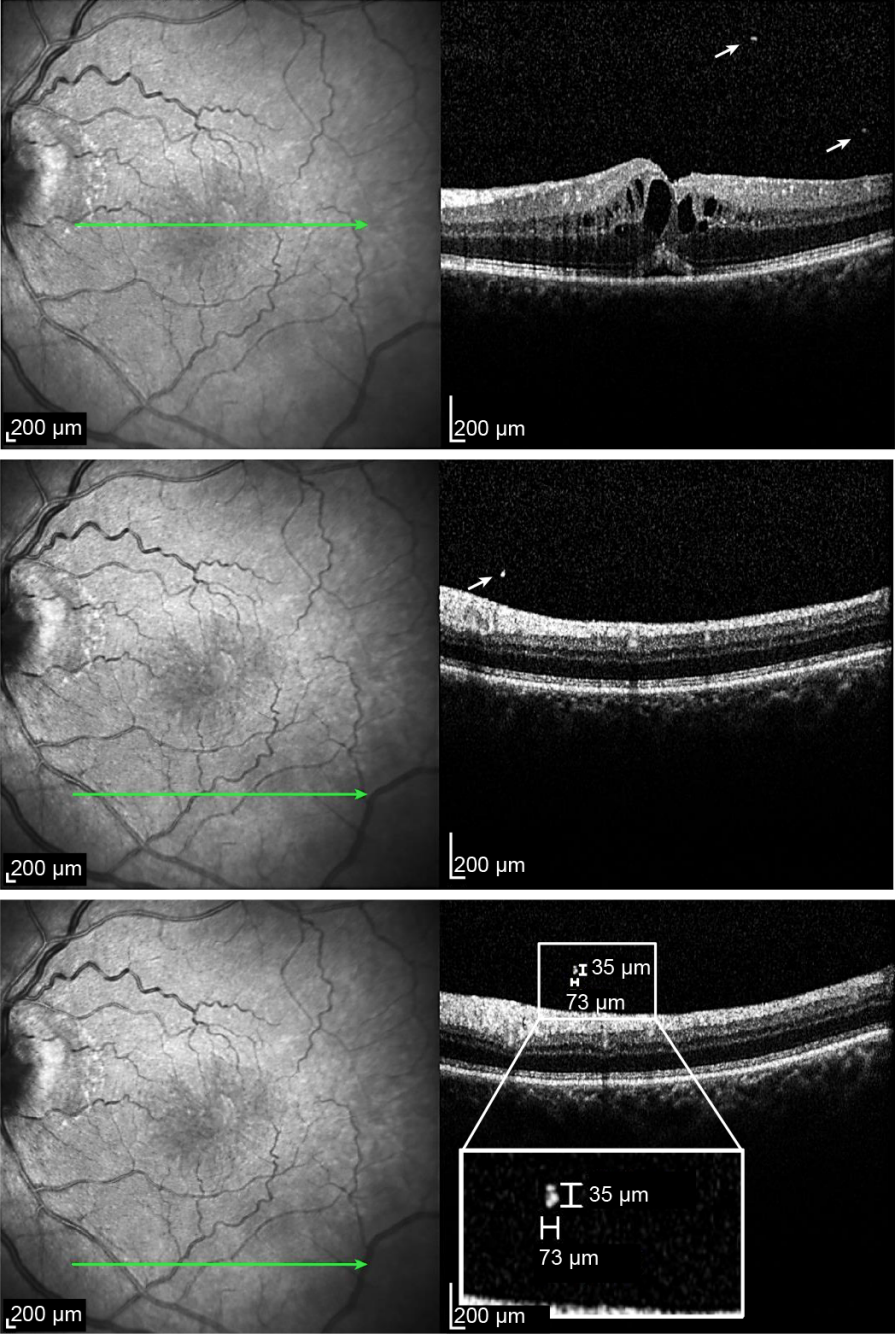
(Upper, Middle) Arrows indicate vitreous hyper-reflective dots (VHDs) in an eye with pseudophakic CME. (Lower) The rectangle displays a magnified VHD with bars measuring its vertical and horizontal diameter. The bar measuring the horizontal diameter appears shorter since the OCT scan is displayed with full vertical resolution (1:1 pixel) instead of the vertical scale adjusted to the horizontal scale (1:1 μm).

Postoperatively, all of the patients received a topical therapy with combined betamethasone and neomycin (Betnesol N®, Tubliux Pharma SpA, Pomezia, Italy) five times a day in tapered frequency for 5 weeks. All of the enrolled patients received an ophthalmic examination including BCVA measurement, fundus examination with dilated pupil and OCT. The OCT image included central subfield thickness (CST) in μm and cube volume (CV) in mm^3^. The CV is a 6-mm volume grid according to the ETDRS, which is automatically calculated with the built-in software by means of the formula surface x thickness [14,22]. In addition, VHDs, defined as clearly visible hyper-reflective dots of variable size of >20 μm in diameter, were assessed (Fig 1) [8]. Total number of VHDs was determined by summing the VHDs of each section. The size of each VHD was defined as the mean of its horizontal and vertical diameter. The diameters were manually assessed using the measurement bars provided by the OCT software (Fig 1). To facilitate statistical analysis, the arithmetic average of all mean sizes of VHDs was calculated. The number of VHDs and their size was assessed by two examiners (WG, DI). The eyes with CME were treated primarily with peribulbar injection of 7 mg betamethasone (Diprophos® 1 ml suspension containing 5 mg betamethason as dipropionate and 2 mg bethamethason as disodium pyrophosphate, Merck Sharp & Dohme GmbH, Vienna, Austria) according to the standard protocol at our hospital. Additional therapy with topical non-steroidal antiphlogistic drops and/or intravitreal cortisone injections in case of persisting CME was given at the physicians’ discretion.

The descriptive data are presented as the mean±standard deviation (range). Normal distribution was assessed with Kolmogorov-Smirnov test. The differences in the continuous data were calculated with the independent t-test or Mann-Whitney-U test depending on the data distribution. Intra- and inter-observer differences regarding the number of VHDs were assessed with intraclass correlation coefficients and 95% confidence intervals (CIs). Correlations between various parameters were determined by using Spearman correlation analysis. The significance of differences between groups in categorical variables was assessed with Fisher's exact test. To identify potential risk factors for CME, logistic regression analysis was performed. The statistics were two-tailed. The threshold for significance was defined as p<0.05.

## Results

Overall, 284 eyes of 267 patients were analyzed. Among them, 165 eyes of 159 patients were excluded due to capsule rupture (n = 8), previous interventions including vitrectomy (n = 27), glaucoma surgery (n = 4) and Yag laser capsulotomy (n = 7); exudative or dry age-related macular degeneration (n = 15); presence of diabetes (n = 17); history or presence of retinal vein occlusion (n = 12) or uveitis (n = 13); macular pucker (n = 25); inappropriate quality of OCT images (n = 13); or incomplete data (n = 12). Twelve other eyes were excluded since CME occurred after >3 months postoperatively. Therefore, 119 eyes of 108 patients were considered for analysis; 56.5% (n = 61) of patients were female, and 43.5% (n = 47) were male. The mean age of the patients was 72.4±10.3 years (43–89). The study group and the control group comprised 63 eyes and 56 eyes, respectively. The patients in the study group were slightly older than those in the control group (Table 1), but this difference did not reach statistical significance.

**Table 1:** Preoperative and postoperative data of the eyes of enrolled subjects.

	Study group (n = 63)	Control group (n = 56)	p value
Age, mean±SD (range)	73.8±10.2 (43–89)	70.8±10.3 (45–87)	0.084^a^
Preoperative keratometric value in diopters, mean±SD (range)	43.7±1.6 (39.4–47.0)	43.3±1.3 (40.9–46.4)	0.169^b^
Preoperative anterior chamber depth in mm, mean±SD (range)	3.1±0.5 (2.1–4.7)	3.1±0.5 (1.9–4.9)	0.571^a^
Preoperative axial length in mm, mean±SD (range)	23.1±1.2 (20.9–27.1)	23.3±1 (20.8–26.6)	0.137^a^
Postoperative CST in μm, mean±SD (range)	515.7±97.8 (304–780)	273.9±18.3 (229–299)	<0.001^a^
Postoperative CV in mm^3^, mean±SD (range)	9.9±0.6 (8.9–11.5)	8.7±0.5 (7.2–9.8)	<0.001^b^
Postoperative BCVA in Snellen lines, mean±SD (range)	0.4±0.2 (0.16–0.8)	0.9±0.1 (0.6–1.0)	<0.001^a^

SD = standard deviation, BCVA = best corrected visual acuity, CST = central subfield thickness, and CV = cube volume.

^a^ Mann-Whitney U test

^b^ independent t-test.

CME was diagnosed 6.2±2.9 weeks (2–12) after surgery, and the follow-up period in the control group averaged 6.6±3.2 weeks (4–12). Preoperative parameters including mean keratometric values, anterior chamber depth, and axial length were comparable among the groups (Table 1). In the study group, the postoperative parameters including CST and CV were significantly increased and the BCVA was consistently and significantly worse (Table 1). Pseudoexfoliation syndrome was found in 5 study eyes (7.9%) and 7 control eyes (12.5%, p = 0.121). The quality score of OCT images averaged 26.6±2.6 (20–32).

The inter- and intra-observer intraclass coefficients for the number of VHDs were 0.969 (95% CI = 0.956–0.979, p<0.001) and 0.989 (95%CI = 0.979–0.990, p<0.001), respectively. Hence, the findings between the observers were consistent. The VHD assessment including its number and size measured by DI (senior author) were considered for analysis. Overall, VHDs were found in 54 study eyes (85.7%) and 21 control eyes (37.5%, p = 0.013). The mean number of VHDs was 3.9±3.4 (0–16) in the study group and 0.7±1 (0–4) in the control group (p<0.001). The mean size of VHDs was 33.5±9.1 μm (21–62) and 36.6±17.9 μm (22–82) in the study and control group, respectively (p = 0.978). The number of VHDs showed no correlation with the duration of follow-up period in either the study group (r = 0.114, p = 0.372) or the control group (r = 0.087, p = 0.523).

Independently of group affiliation, the number of VHDs showed a correlation with CST (r = 0.584, p<0.001) and CV (r = 0.525, p<0.001) and a negative correlation with postoperative BCVA (r = -0.563, p<0.001). The size of VHDs had no correlation with CST (r = 0.063, p = 0.589), CV (r = 0.07, p = 0.553) or postoperative BCVA (r = -0.04, p = 0.742). The number of VHDs and age >70 years were identified as relevant risk factors for CME, with an odds ratio (OR) of 2.406 (95% CI 1.687–3.431, p<0.001) and 2.815 (95% CI 0.972–8.115, p = 0.056), respectively. Other assessed factors including age in general (OR 1.043, 95% CI 0.990–1.098, p = 0.116), mean keratometric value (OR 1.200, 95% CI 0.807–1.783, p = 0.368), anterior chamber depth (OR 1.336, 95% CI 0.396–4.503, p = 0.641), axial length in general (OR 0.895, 95% CI 0.528–1.518, p = 0.681), axial length <23 mm (OR 3.358, 95% CI 0.738–15.275, p = 0.117) and pseudoexfoliation syndrome (OR 0.977, 95% CI 0.193–4.954, p = 0.915) showed no significant associations with CME.

The mean number of peribulbar steroid injections was 1.4±0.7 (1–4). The frequency of peribulbar steroid injections did not correlate with the number or size of VHDs; however, it significantly correlated with CST (Table 2).

**Table 2:** Correlation coefficient r and p value estimated with Spearman’s correlation between frequency of peribulbar steroid injections or intravitreal steroid injections and relevant Clinical parameters.

	Peribulbar steroid injection	Intravitreal steroid injection
Number of VHDs, r (p)	0.157 (0.219)	0.101 (0.432)
Size of VHDs, r (p)	0.08 (0.564)	0.093 (0.47)
Postoperative CST, r (p)	0.258 (0.041)	0.223 (0.079)
Postoperative CV, r (p)	0.251 (0.068)	0.210 (0.098)

VHDs = vitreous hyper-reflective dots, CST = central subfield thickness, and CV = cube volume.

In 4 eyes (6.3%), 4 mg triamcinolone acetonide (Triescence, Alcon Laboratories, USA) was additionally administered intravitreally, and in 1 eye (1.6%), 700 μg dexamethasone (Ozurdex, Allergan, Ireland) was additionally administered intravitreally. There was no correlation between the application of intravitreal steroid injections and the number or the size of VHDs (Table 2). Further, there was no statistically significant correlation between intravitreal steroid injections and postoperative CST and CV (Table 2). Thirty-two study eyes (50.8%) were additionally treated with topical nonsteroidal antiphlogistic drops.

## Discussion

Our study showed that after uneventful phacoemulsification VHDs were detected more frequently in eyes with CME (85.7%) than in those without CME (37.5%). This difference was statistically significant. Moreover, in eyes with CME, the mean number of VHDs was significantly higher than in those with no CME. Generally, the number of VHDs had an impact on the extent of the macular thickening. In detail, there was a significant correlation between the number of VHDs and CST, CV and correspondingly the postoperative BCVA. Our study thus confirms the association between the presence of VHDs and CME, as recently reported by Oh et al. [8]. In their longitudinal study, VHDs observed 1 week after an uneventful phacoemulsification were highly predictive of the development of CME 1 month after surgery [8]. In contrast to their study, we could only assess the existence of VHDs in eyes that already showed CME since no consecutive follow-up OCT examinations after the surgery were available. In addition to the presence of VHDs, age was previously shown to be a predictive factor for CME [8]. In our study, however, the age in general did not considerably increase the risk for CME, but advanced age, defined as >70 years, was identified as a relevant risk factor. The axial length was also shown to be an important risk factor. Although it was not identified as predictive for CME, eyes with shorter axial length showed a higher prevalence of VHDs [8]. In our study, no correlation between the number of VHDs and axial length was found; however, in the regression analysis, axial length in general had an OR of 0.895, indicating that increasing axial length reduced the risk for CME. In addition, shorter eyes, defined as axial length <23 mm, were found to be at higher risk for CME, with an OR of 3.358. However, this association was not statistically significant. Other assessed factors including male gender, mean keratometric value, anterior chamber depth, and pseudoexfoliation syndrome were not significantly associated with CME occurrence. The size of VHDs did not correlate with CST or CV in our study. However, the size of VHDs was assessed by taking the arithmetical mean of all of the detected VHDs to facilitate the statistical evaluation, and this procedure might have obscured any effect of size.

The frequency of peribulbar steroid injections or the need for intravitreal steroid injections did not correlate with the number or the size of VHDs (Table 2). We could only find a significant correlation between the extent of CME and the frequency of peribulbar steroid injections. This finding indicates that eyes with a greater extent of CME more likely need repeated steroid therapy. The lack of a correlation between the number of VHDs and the frequency of peribulbar or intravitreal steroid injections may indicate that VHDs do not predict the responsivity of CME to steroid treatment. However, several confounding factors make it impossible to draw this conclusion. First, 50.8% of eyes received topical nonsteroidal antiphlogistic drops additionally to peribulbar or intravitreal steroid injections. Since topical non-steroidal antiphlogistic drops are a key element in the management of patients with pseudophakic CME, it certainly altered our results. We did not evaluate the impact of topical non-steroidal antiphlogistic drops on the CME resolvement since this would be beyond the scope of our study. Second, we only had follow-up OCT scans of eyes with non-resolving CME. Follow-up OCT scans of all eyes would have provided more conclusive evidence in this regard. Unfortunately, in our unit follow-up OCT examinations are performed routinely only in cases with non-resolving CME.

The exact nature of VHDs is not fully understood since no histopathological evaluations exist. Several theories explain their potential origin. First, VHDs may represent lens fragments formed by phacoemulsification that are accelerated through the zonula into the vitreous cavity [18]. A previous report revealed that 16.6% of eyes showed retrocapsular lens fragments intraoperatively as a result of phacoemulsification [18]. The only risk factor for the occurrence of retrocapsular fragments was the equivalent phaco time, defined as a product of phaco power and phaco time [18]. Unfortunately, this parameter was not assessable in our study. Second, hyper-reflective spots were also identified with OCT in the vitreous of patients with posterior uveitis representing a conglomeration of inflammatory cells [19]. Therefore, VHDs might represent conglomerated inflammatory cells also in our study. In this case, VHDs would be a consequence of the inflammatory reaction after surgery rather than a factor contributing to the inflammation process. However, hyper-reflective spots consisting of conglomerations of inflammatory cells had a size of less than 20 μm [19]. In contrast, we only considered VHDs >20 μm to achieve a better comparability to the study of Oh et al. [8]. Third, epithelial cells from the ciliary body might be released during phacoemulsification and float into the vitreous cavity [20]. The limitations of this theory are that epithelial cells are commonly smaller than 20 μm and the inflammatory potential of released epithelial cells is questionable [20]. Fourth, VHDs might be vitreous collagen fibrils that are denaturated by the heat of the phacoemulsification tip [21]. This hypothesis is based on the observation that a continuous-wave carbon-dioxide laser beam induces heat-denaturation of collagen fibrils in the bovine and porcine vitreous [21]. However, we used no laser, and we enrolled only eyes with intact posterior capsule. In our opinion, VHDs most likely represent lens fragments since lens material is known to provoke intraocular inflammation [24]. Furthermore, the prevalence of retrocapsular VHDs after cataract surgery in 16.6% of eyes is comparable to the overall occurrence of pseudophakic CME in 13.9% of eyes [8,18]. Oh et al. reported about the presence of VHDs in the preoperative OCT in 2.7% of eyes [8]. Their assumed nature is aggregation of collagen fibrils or asteroid bodies measuring less than 20 μm in diameter which is below the threshold defined for VHDs [8]. In their study they did not find a correlation between preoperatively detected VHDs and the occurrence of CME [8]. However, the limitation of this conclusion is that the commonly poor quality of preoperative OCT images compromised the exact assessment of number and size of preoperative VHDs. Therefore it remains unknown if some of the postoperatively detected VHDs might have preexisted [8]. Unfortunately, we could not evaluate the relevance of these preoperative VHDs in our study, because no preoperative OCT scans were available in the vast majority of patients.

This study has some limitations. A higher number of eyes would likely reveal the impact of shorter axial length on the occurrence of CME and the correlations between the application of intravitreal steroid injection and postoperative CST and CV at statistically significant levels. A prospective design with preoperative and follow up scans would have more conclusively assessed the role of preoperatively detected VHDs on the development of CME or a potential association between the number of VHDs and the responsivity of CME to the steroid therapy. Furthermore, our study can only presume but not verify the origin of VHDs. Histopathological examination of VHDs is critical since performing vitrectomy for this purpose in otherwise healthy eyes would be of ethical concern. Further, we were only able to assess detectable VHDs in the preretinal vitreous. Thus, VHDs beyond the detection range of OCT and those between the sections of the OCT image remained undetected. This technical limitation is a known bias [8].

In conclusion, a significantly higher prevalence and number of VHDs is noted in eyes with pseudophakic CME compared to eyes with no CME following phacoemulsification. An increased number of VHDs was associated with a greater extent of macular thickening. No association was found between the number of VHDs and the number of received periabulbar or intravitreal steroid injections.

## Supporting information

S1 Data Collected patient data. (XLSX)
